# Protocatechualdehyde Rescues Oxygen-Glucose Deprivation/Reoxygenation-Induced Endothelial Cells Injury by Inducing Autophagy and Inhibiting Apoptosis *via* Regulation of SIRT1

**DOI:** 10.3389/fphar.2022.846513

**Published:** 2022-03-31

**Authors:** Shidong Cao, Senmiao Chen, Xilin Qiao, Yan Guo, Fang Liu, Zhishan Ding, Bo Jin

**Affiliations:** ^1^ School of Life Science, Zhejiang Chinese Medical University, Hangzhou, China; ^2^ School of Basic Medicine, Zhejiang Chinese Medical University, Hangzhou, China; ^3^ School of Medical Technology and Information Engineering, Zhejiang Chinese Medical University, Hangzhou, China

**Keywords:** protocatechualdehyde, autophagy, apoptosis, SIRT1, HUVEC, OGD/R

## Abstract

**Background:** Oxidative stress-induced endothelial cell death, such as apoptosis and autophagy, plays a critical role in ischemia-reperfusion injury. Protocatechualdehyde (PCA) is a major bioactive component of the traditional Chinese medicine *Salvia miltiorrhiza Bunge* (Lamiaceae), and it has been proved to be effective in the prevention and treatment of ischemic cardiovascular and cerebrovascular diseases. However, its role in oxidative stress-induced endothelial cell death and its underlying mechanisms remains unclear. This study aims to investigate the effects and mechanisms of PCA on endothelial cell apoptosis and autophagy induced by oxygen-glucose deprivation/reoxygenation (OGD/R) injury.

**Methods:** After OGD/R induction, human umbilical vein endothelial cells (HUVECs) were treated with different concentrations of PCA. Cell viability, apoptosis, and autophagy were detected by Cell Counting Kit-8 assay, flow cytometry, and monodansylcadaverine assay, respectively. Western blot was applied to explore the effects of PCA on the expression levels of relevant protein factors.

**Results:** The results show that PCA significantly promoted cell survival rate and cell proliferation and enhanced the antioxidant activity in OGD/R-induced HUVECs. PCA inhibited HUVECs apoptosis, as evidenced by decreased expression of cleaved-caspase-3, Bcl2-associated X (BAX), and increased expression of Bcl-2. PCA induced autophagy by reducing the expression of P62 while increasing the expression of Beclin-1 and LC3 II/I. Meanwhile, PCA enhanced the expression of Sirtuin 1 (SIRT1) and suppressed the expression of P53. When SIRT1 was inhibited by selisistat or SIRT1 small-interfering RNA, the anti-apoptotic and pro-autophagy abilities of PCA were attenuated.

**Conclusion:** These results demonstrated that PCA rescued HUVECs from OGD/R-induced injury by promoting autophagy and inhibiting apoptosis through SIRT1 and could be developed as a potential therapeutic agent against ischemic diseases.

## Introduction

Ischemic-reperfusion (IR)-induced vascular cell death, which induces dysregulation of vascular reactivity, is a key contributor to mortality and morbidity in ischemic diseases, including myocardial IR injury, cerebral IR injury, and postoperative IR injury (Pan et al., 2021; Zhang et al., 2021). Endothelial cells (ECs) line the intimal layer of blood vessels and play an important role in maintaining vascular homeostasis. Ischemic-related oxidative stress-induced dysfunction of ECs and cell death are considered to be the initiating events during IR injury (Kurian et al., 2016; Amruta and Bix, 2021; He et al., 2021). Thus, rescuing of ECs from IR damage emerges as a potential therapeutic strategy for ischemic diseases such as myocardial infarction, ischemic stroke, and organ transplantation (Amruta and Bix, 2021; Li et al., 2022). Multiple cell death pathways are involved in the pathogenesis of endothelial dysfunction, including apoptosis, autophagy, necroptosis, ferroptosis, and pyroptosis (Tuo et al., 2022). However, it remains unclear whether there is only one or multiple pathways that are particularly significant in this context. Oxidative stress-induced apoptosis is the most common form of cell death in endothelial dysfunction (Zhang and Li, 2020). In addition, induction of pyroptosis and disruption of autophagy also play a critical role in endothelial dysfunction (Peng et al., 2021; Guo et al., 2022).

Protocatechualdehyde (PCA) is a main phenolic acid in the root of *Salvia miltiorrhiza Bunge* (Lamiaceae), a traditional Chinese medicine widely used in the treatment of cardiovascular diseases and ischemic stroke (Ji et al., 2000; Guo et al., 2022). PCA has been reported to have many pharmacological uses, such as an antioxidant (Guo et al., 2017) and anti-apoptosis (Wan et al., 2021). In addition, studies have confirmed the potential protective effect of PCA on ECs (Xing et al., 2012). Ji et al. (2021) reported that PCA can prevent oxidative stress and restore endothelial function by improving oxidative inactivation of vascular nitric oxide in diabetic conditions. Our previous data also demonstrated that PCA can protect ECs from excessive autophagic damage induced by oxidative stress (Guo et al., 2021) and prevent oxygen-glucose deprivation/reoxygenation (OGD/R)-induced pyroptosis of microvascular ECs in the rat brain (Guo et al., 2022). However, the precise protective effects of PCA on endothelial cell autophagy and apoptosis, and the molecular mechanisms at cellular level, have not been reported. Herein, in this study, an investigation focused on the role of PCA in apoptosis and autophagy to explore whether it could rescue human umbilical vein endothelial cells (HUVECs) from OGD/R-induced injury was undertaken.

## Materials and Methods

### Materials

**FIGURE 1:**
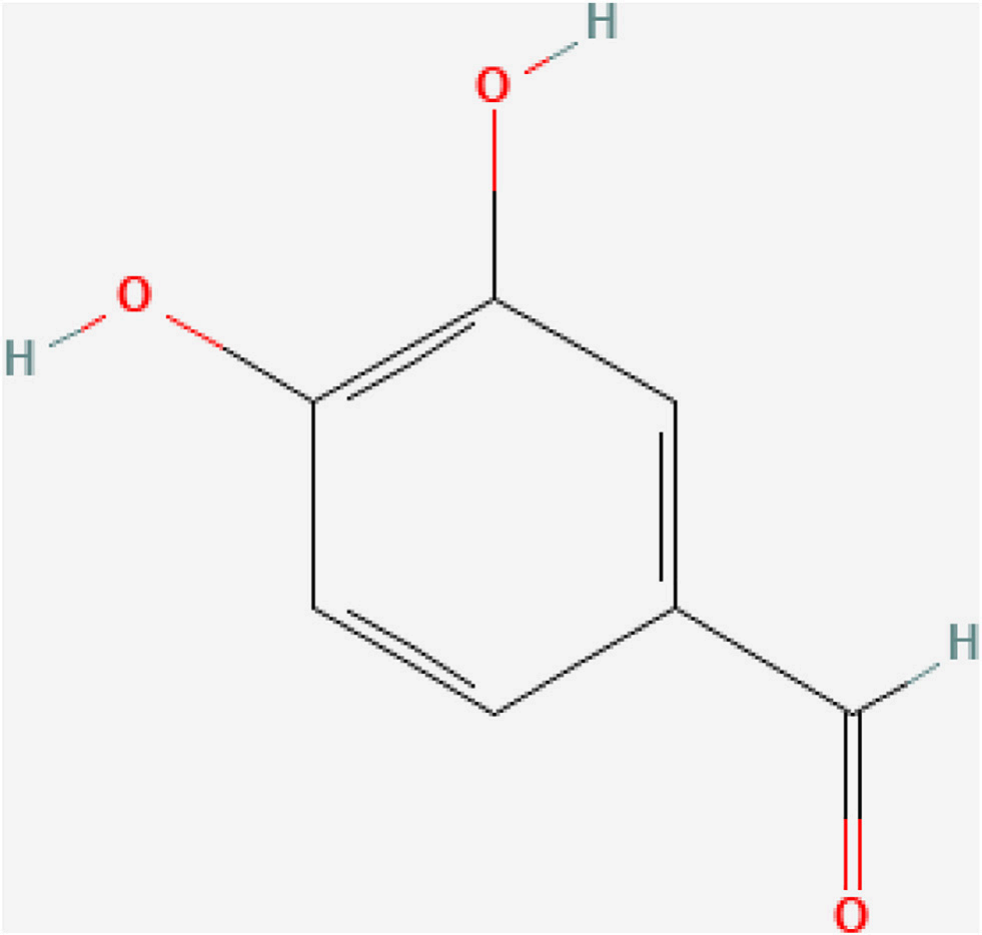
The chemical structure of protocatechualdehyde.

PCA (C_7_H_6_O_3_, purity ≥ 99% High-Performance Liquid Chromatography) was purchased from Aladdin Company (Shanghai, China) (Figure 1). HUVECs were purchased from the Cell Bank of Type Culture collection of the Chinese Academy of Sciences (Shanghai, China).

### Cell Cultures

HUVECs were cultured with Royal Park Memorial Institute (RPMI) 1640 Medium (Gibco, Grand Island, NY, United States) supplemented with 10% fetal bovine serum (Tianhang, Hangzhou, China), 100 U/mL penicillin, and 100 μg/ml streptomycin (Gibco, Grand Island, NY, United States) at 37°C in a humidified atmosphere of air containing 5% CO_2_. All experiments were strictly conducted in cells between the fourth and seventh generations.

### OGD/R Model and Cell Treatment

OGD/R model was carried out as described previously (Guo et al., 2022). Briefly, HUVECs were cultured in glucose-free and serum-free Minimum Essential Medium (Gibco, Grand Island, NY, United States) within an anaerobic chamber containing 1% O_2,_ 5% CO_2_, and 94% N_2_ for 10 h at 37°C. After hypoxia, cells were incubated with full RPMI 1640 Medium without or with PCA at given concentrations for 24 h. When selisistat (EX527) was used to inhibit the activity of Sirtuin 1 (SIRT1), EX527 (10 μM) (Beyotime, Shanghai, China) was added to full RPMI 1640 Medium and incubated 2 h before OGD/R treatment (Deng et al., 2021).

### Cell Viability Assay

Cell viability was evaluated using the Cell Counting Kit-8 (CCK-8) assay (Beyotime, Shanghai, China) following the manufacturer’s protocol. HUVECs (5000 cells/well) were seeded in 96-well microliter plates and incubated with PCA according to different experimental purposes. After different treatments, 10 μL of CCK-8 solution was added to each well and incubated for 2 h at 37°C. The absorbance at 450 nm was measured with a microplate reader (Bio Tek, Winooski, VT, United States). Cell viability was defined as a percentage of the control group.

### Cell Proliferation Assay and Lactate Dehydrogenase Assay

The 5-ethynyl-2′-deoxyuridine (EDU) assay (Beyotime, Shanghai, China) was used to indicate cell proliferation. After different treatment, cells were incubated with 10 mM EDU medium diluent at 37°C for 2 h, 200 μL of click staining reaction solution was added and incubated for 30 min in the dark, and 200 μL of 1 × Hoechst 33342 reaction solution was incubated for 10 min. After washing three times with phosphate-buffered saline (PBS), five visual fields were randomly selected under the fluorescence microscope (Nikon, Tokyo, Japan) for observation.

The cytotoxicity of PCA was examined by LDH assay. After treatment, the media was collected and detected using a commercial LDH assay kit (Beyotime, Shanghai, China) according to the manufacturer’s instructions. The absorbance at 490 nm was measured and the relative LDH level was calculated.

### Detection of Reactive Oxygen Species and Superoxide Dismutase

Following the manufacturer’s instructions (Beyotime, Shanghai, China), the HUVECs were collected and washed three times with cold PBS. Then, the cells were incubated with the ROS detection work solutions (10 μM) at 37°C for 20 min in the dark. The relative fluorescence intensity of the cells was measured using the fluorescence microscope.

The concentration of SOD in HUVECs was determined using a commercial kit (Beyotime, Shanghai, China). After treatment, cell lysates were prepared, and the protein concentrations were measured using the bicinchoninic acid (BCA) protein assay kit (Biosharp, Hefei, China). SOD levels were quantified according to the manufacturer’s protocols.

### Apoptosis Detection With Flow Cytometry

Flow cytometry was used to detect cell apoptosis. Briefly, cells were harvested and stained with Fluorescein isothiocyanate-conjugated anti-Annexin V antibody and PI (BD Biosciences, Franklin Lakes, NJ, United States) for 15 min under darkness. Then apoptotic cells were analyzed using flow cytometry (BD Biosciences, Franklin Lakes, NJ, United States).

### Monodansylcadaverine Staining Assay

MDC fluorescent staining kit (Beyotime, Shanghai, China) was used to detect the level of autophagy occurring in cells. After different treatments, cells were harvested and incubated with 1 ml MDC staining solution for 30 min at 37°C in the dark. After washing three times with washing solution, cells were resuspended in the collection buffer, and assayed under the flow cytometry (Beckman coulter, Brea, CA, United States).

### SIRT1 Activity Analysis

SIRT1 activity was measured by an enzyme-linked immunosorbent assay kit (Lengton Bioscience, Shanghai, China). After treatment, the cell culture supernatant was collected with each procedure following the manufacturer’s instructions. SIRT1 activity was quantified according to the manufacturer’s instructions.

### Small-Interfering RNA Transfection

SIRT1 siRNA (Santa Cruz Biotechnology, Beijing, China) were transfected into HUVECs by using Lipofectamine™ 3000 Transfection reagent (Invitrogen, Carlsbad, CA, United States) according to the manufacturer’s protocols. After 6 h of transfection, the transfection medium was replaced with normal RPMI 1640 medium and incubated for 24 h, followed by OGD/R treatment.

### Western Blot Analysis

Total protein was extracted with radioimmunoprecipitation assay lysis buffer (Beyotime, Shanghai, China), and quantified using a BCA protein assay kit. Protein samples were analyzed by sodium dodecyl sulfate-polyacrylamide gel electrophoresis and transferred to polyvinylidene fluoride membranes (Millipore, Shanghai, China). After blocked with 5% non-fat silk milk at room temperature for 1 h, membranes were incubated with primary antibodies at 4°C overnight. Then the membranes were incubated with horseradish peroxidase-conjugated secondary antibodies at room temperature for 2 h. Finally, immunoblotting bands were visualized with Chemi Scope 6200 Touch image capture system (Clinx, Shanghai, China), following reaction with an electrochemiluminescence detection kit (Biosharp, Hefei, China). Western blot data were processed with ImageJ software (National Institutes of Health, Bethesda, MD, United States). β-actin was used as the loading control.

Antibodies and dilutions: anti-SIRT1 (60303-1-lg, 1:2,000), anti-LC3 (14600-1-AP, 1:2,000), anti-P62 (66184-1-ig, 1:1,000) were purchased from Proteintech (Chicago, IL, United States), anti-P53 (YM3052, 1:1,000), anti-Bcl2-associated X (BAX) (YM3619, 1:1,000), anti-Bcl-2 (YM3041, 1:1,000) were purchased from ImmunoWay (Plano, TX, United States), anti-Cleaved-Caspase3 (AF7022, 1:1,000) was purchased from Affinity Biosciences (Beijing, China), anti-Beclin-1 (PD017, 1:1,000) purchased from MBL (Beijing, China), and anti-β-actin (DW9562, 1:5,000), anti-Mouse IgG (DW0990, 1:5,000) and anti-Rabbit IgG (DW-GAR007, 1:5,000) were purchased from Dawen Biotec (Hangzhou, China).

### Statistical Analysis

All data were presented as the mean ± SD calculated by GraphPad Prism5 (GraphPad Software Inc., La Jolla, CA, United States). For differences between groups, one-way analysis of variance, followed by Tukey post hoc test, was performed. *p* < 0.05 was considered statistically significant.

## Results

### PCA Protects HUVECs Against OGD/R Injury

**FIGURE 2:**
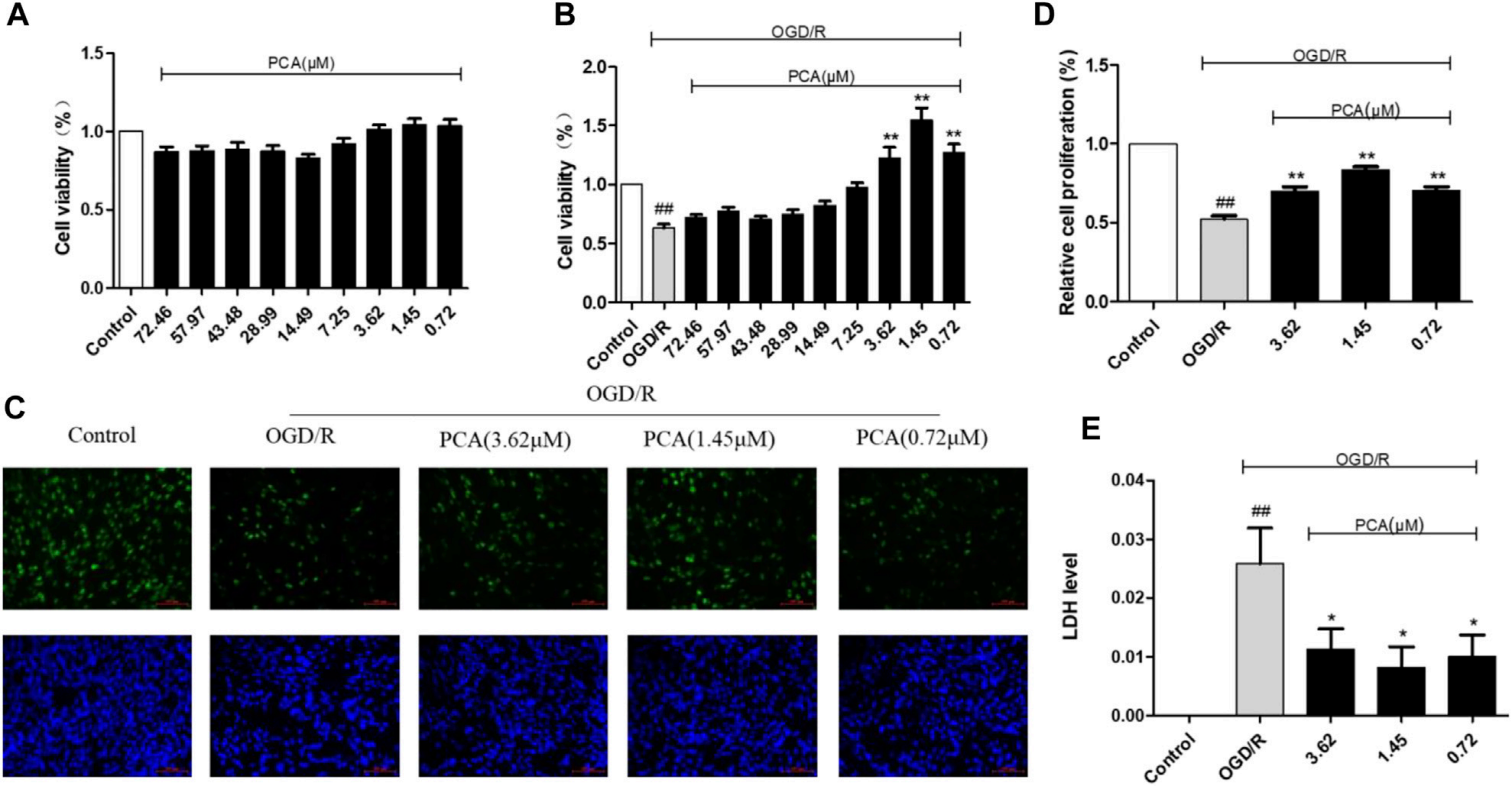
PCA protects HUVECs against OGD/R injury. **(A)** HUVECs were treated with different concentrations of PCA (0.72–72.46 μM) for 24 h, and measured cell viability with CCK-8; **(B)** HUVECs were treated with OGD for 10 h, incubate different concentrations of PCA (0.72–72.46 μM) for 24 h, and measured cell viability with CCK-8. **(C)** The rate of cell proliferation in HUVECs was detected using an EDU cell proliferation assay; **(D)** PCA reverses OGD/R-induced HUVECs cell proliferation rate. **(E)** The relative LDH level in HUVECs was detected using an LDH assay. The values are expressed as the mean ± SD (*n* = 3). ^##^ *p* < 0.01 vs. Control, ^**^ *p* < 0.01, ^*^ *p* < 0.05 vs. OGD/R.

Firstly, the CCK-8 assay results showed that the effect of PCA at 0.72–72.46 μM on cell viability was not significantly different from the control group (Figure 2A), while PCA at 0.72, 1.45, and 3.62 μM significantly improved cell viability after OGD/R injury (*p* < 0.01) (Figure 2B). Therefore, we selected these three concentrations for the subsequent experimental study.

Then, in EDU assay, green fluorescence represented proliferating cells, and all cell nuclear was stained with blue fluorescence. Their ratio indicated cell proliferation. The results (Figure 2C) showed that OGD/R reduced the cell proliferation rate, whereas PCA treatment reversed this effect by significantly increasing the cell proliferation rate (*p* < 0.01) (Figure 2D).

Furthermore, the results of the LDH assay showed that the relative release rate of LDH increased in the cells of the OGD/R model group, but PCA reduced the relative release rate of LDH (*p* < 0.05) (Figure 2E).

### PCA Increases Antioxidant Activity in OGD/R-Induced HUVECs

**FIGURE 3:**
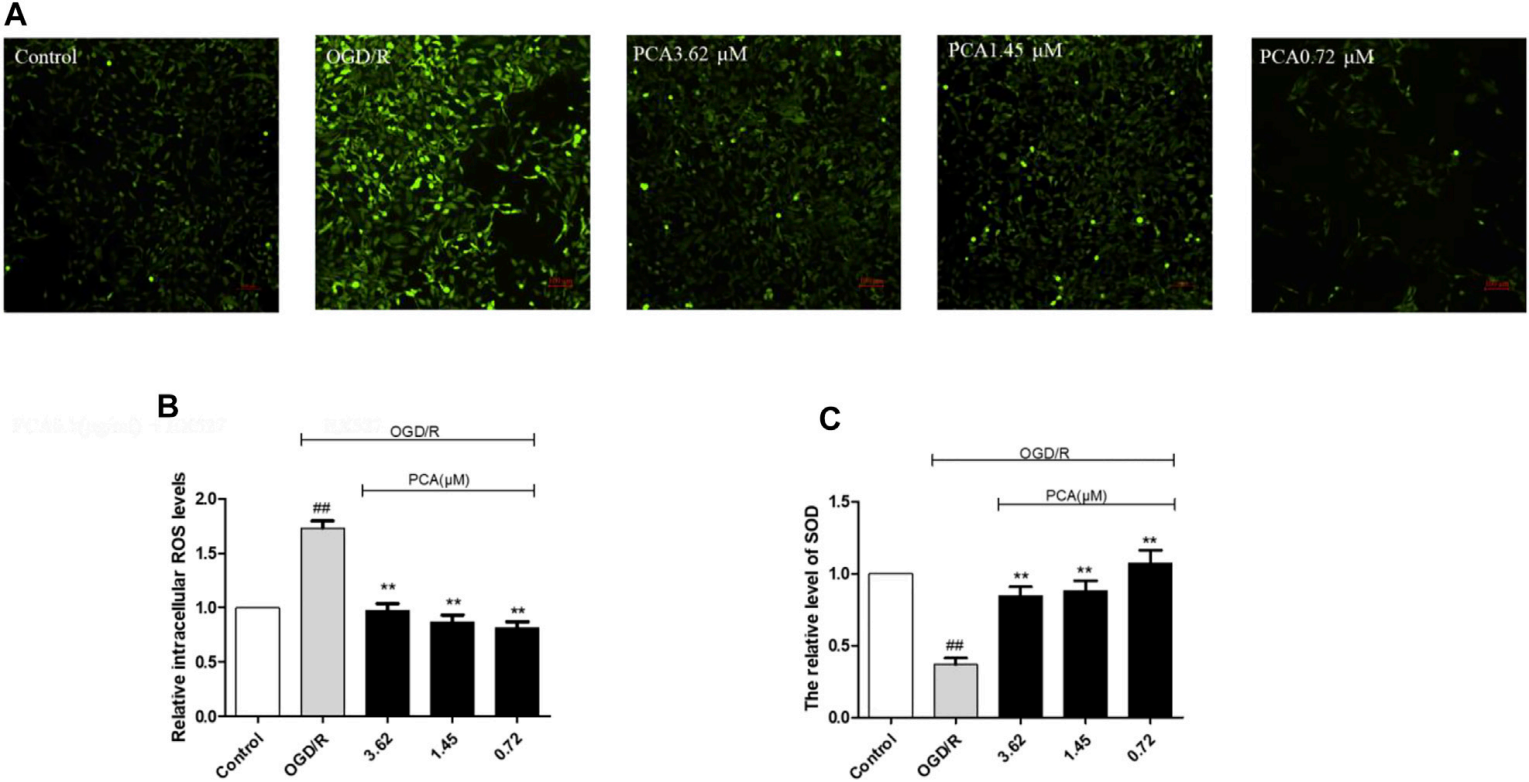
PCA increased antioxidant activity in OGD/R induced HUVECs. **(A, B)** Quantitation of HUVECs ROS level was shown. **(C)** Quantitation of HUVECs SOD activity was shown. The values are expressed as the mean ± SD (*n* = 3). ^##^ *p* < 0.01 vs. Control, ^**^ *p* < 0.01 vs. OGD/R.

The ROS concentration was significantly increased in the OGD/R model group, and PCA treatment significantly reduced the ROS level (Figures 3A,B) (*p* < 0.01).

In addition, compared with the model group, PCA treatment significantly increased SOD levels (*p* < 0.01) (Figure 3C). This indicated that PCA increased antioxidant activity in OGD/R-induced HUVECs.

### PCA Inhibits Apoptosis in OGD/R-Induced HUVECs

**FIGURE 4:**
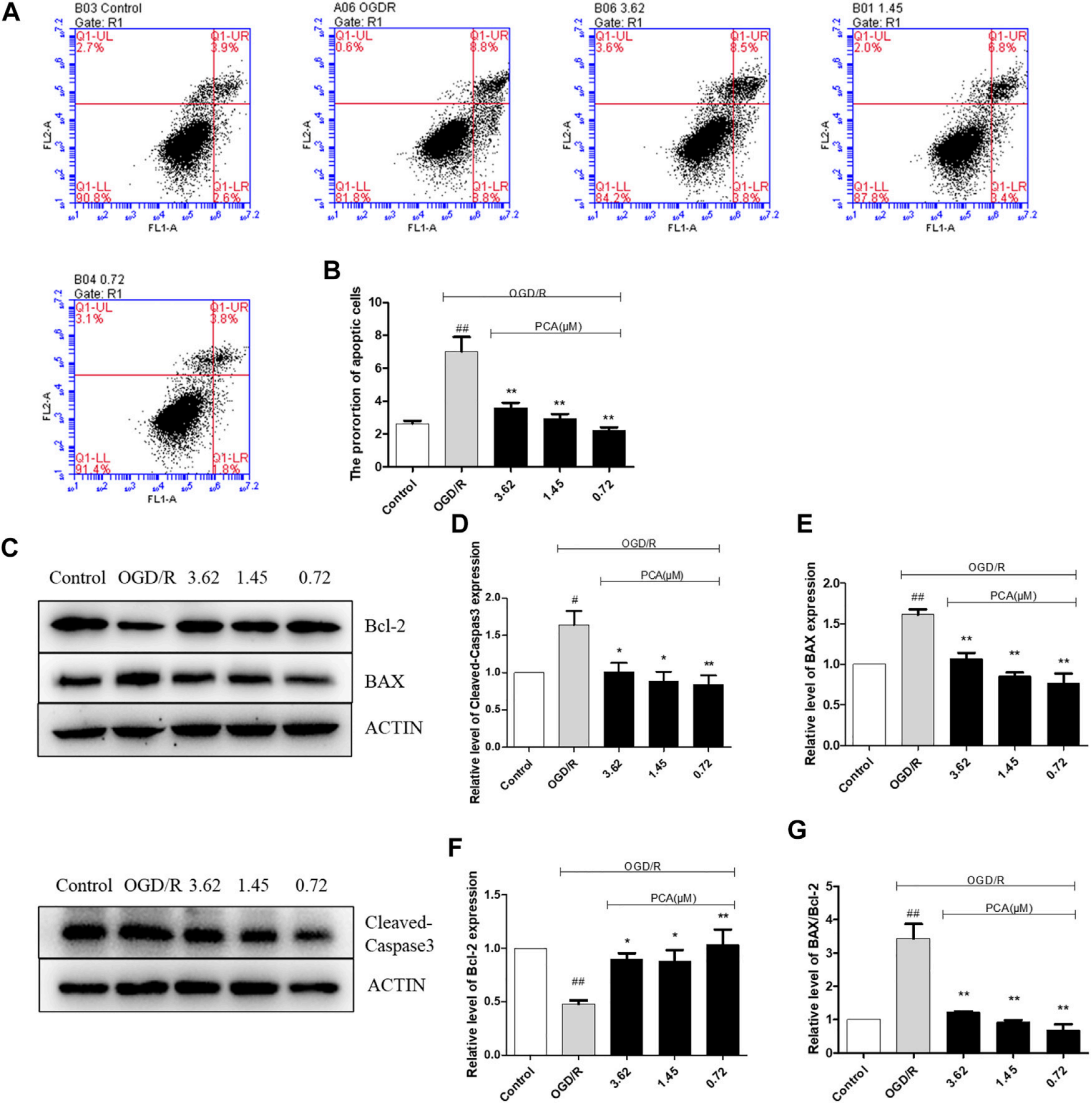
PCA regulated HUVECs apoptosis. **(A, B)** Representative images of flow cytometry analysis of PCA influence and quantification of cell apoptosis rate. **(C–G)** Representative images of WB analysis and the semi-quantification of Cleaved-Caspase3, BAX and Bcl-2 in HUVECs. The values are expressed as the mean ± SD (*n* = 3). ^##^ *p* < 0.01 vs. Control, ^**^ *p* < 0.01, ^*^ *p* < 0.05 vs. OGD/R.

Flow cytometry results are shown in Figures 4A,B. Compared with the control group, the apoptosis rate in the model group was increased (*p* < 0.01), and PCA (0.72, 1.45, and 3.62 μM) attenuated the apoptosis rate (*p* < 0.01).

Apoptosis-related proteins were further measured. As shown in Figures 4C–G, compared with the control group, the expression of Cleaved-Caspase3 and BAX protein in the OGD/R model group was significantly increased (*p* < 0.01), and the expression of Bcl-2 protein was significantly decreased (*p* < 0.01). Compared with the OGD/R model group, PCA (0.72, 1.45, and 3.62 μM) treatment can reverse these effects (*p* < 0.05).

### PCA Promotes Autophagy in OGD/R-Induced HUVECs

**FIGURE 5:**
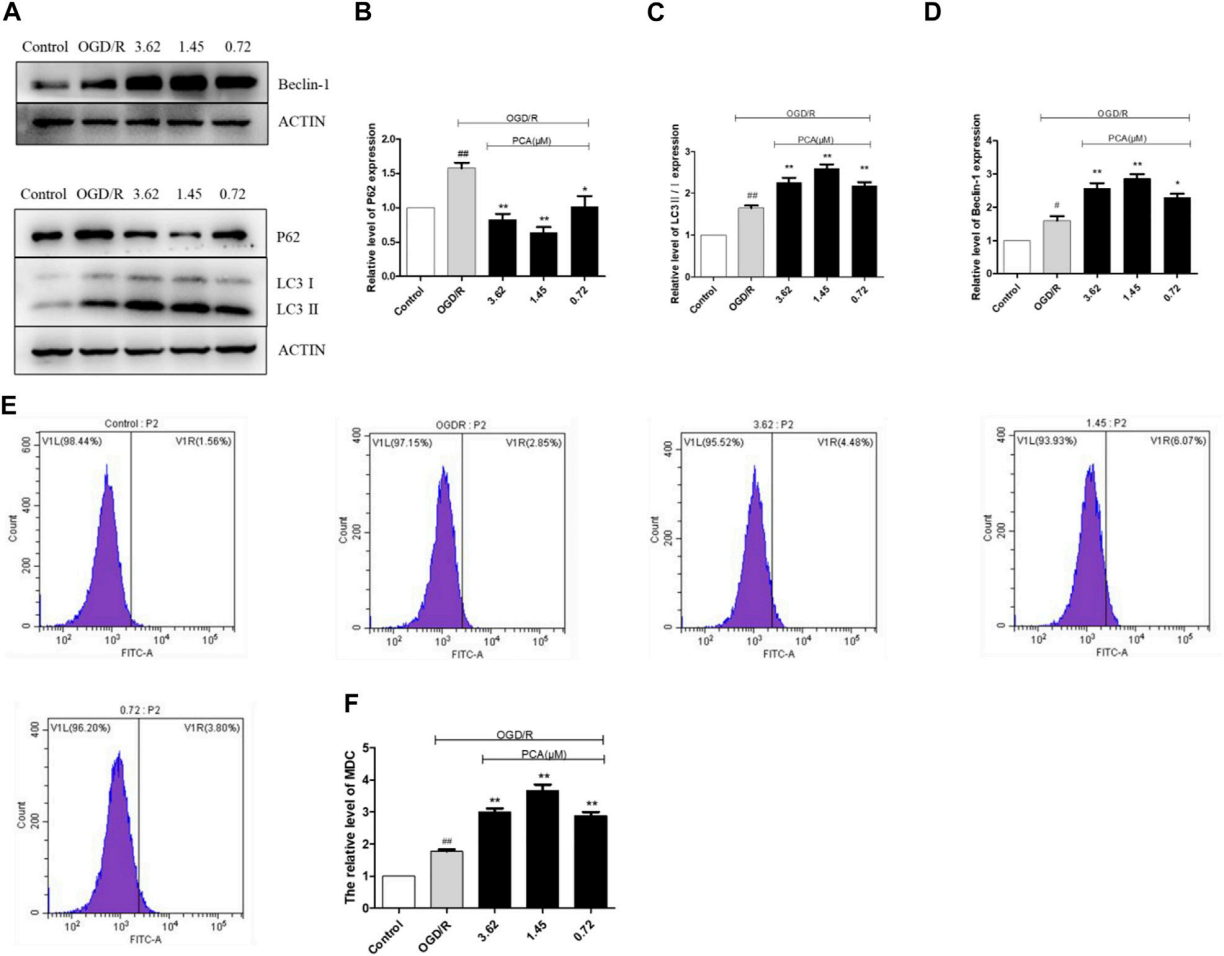
PCA regulated HUVECs autophagy. **(A–D)** Representative images of WB analysis and the semi-quantification of P62, Beclin-1 and LC3 in HUVECs. **(E, F)** Representative images of flow cytometry analysis of PCA influence and quantification of cell autophagy rate. The values are expressed as the mean ± SD (*n* = 3). ^##^ *p* < 0.01, ^#^ *p* < 0.05 vs. Control, ^**^ *p* < 0.01, ^*^ *p* < 0.05 vs. OGD/R.

As presented in Figures 5A–D, compared with the control group, the expressions levels of P62, Beclin-1 and LC3 II/I protein were markedly elevated in the OGD/R group (*p* < 0.01). PCA (0.72, 1.45, and 3.62 μM) treatment further enhanced the level of Beclin-1 and LC3 II/I and reduced the expression of P62 (*p* < 0.01). The MDC staining results indicated that PCA (0.72, 1.45, and 3.62 μM) treatment could increase autophagy levels compared with OGD/R group (*p* < 0.01) (Figures 5E,F).

### The Effect of PCA on HUVECs OGD/R Injury Involved the Regulation of SIRT1

**FIGURE 6:**
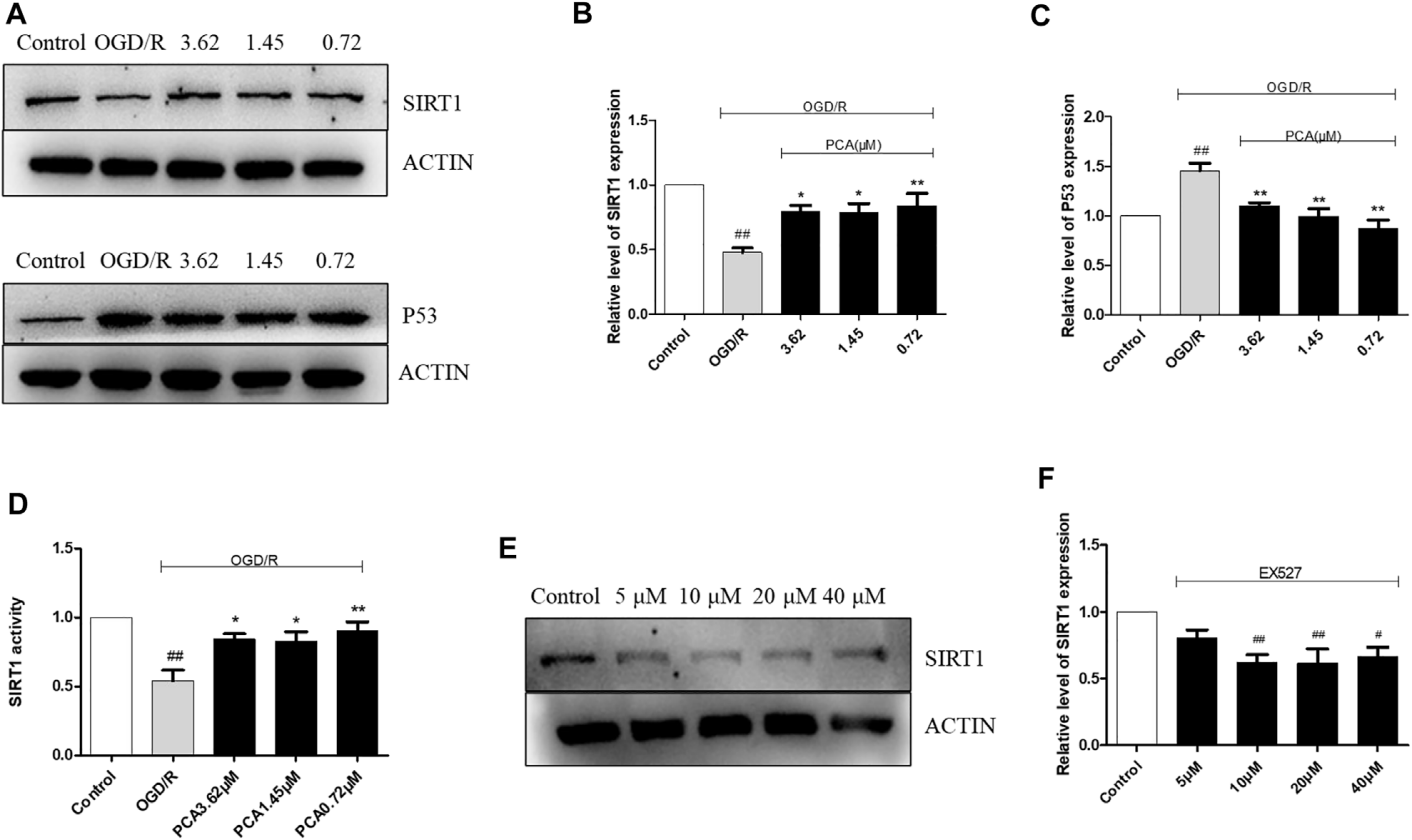
Effects on SIRT1 and P53 expression and activity. **(A–C)** Representative images of WB analysis and the semi-quantification of SIRT1, P53 in HUVECs. **(D)** PCA can increase SIRT1 activity after OGD/R-induced injury **(E, F)** Expression levels of SIRT1 proteins under different concentrations of EX527 (5–40 μM). The values are expressed as the mean ± SD (*n* = 3). ^##^ *p* < 0.01, ^#^ *p* < 0.05 vs. Control, ^**^ *p* < 0.01, ^*^ *p* < 0.05 vs. OGD/R.

The results of SIRT1 and P53 expression levels are shown in Figures 6A–C. Compared with control group, OGD/R induction decreased the expression level of SIRT1 and enhanced the level of P53 (*p* < 0.01), but PCA (0.72, 1.45, and 3.62 μM) treatment reversed these effects (*p* < 0.05). The MDC results further indicated that PCA had the same promoting effect on SIRT1 activity (*p* < 0.05), and the effect of PCA 0.72 μM was more pronounced than others (Figure 6D).

**FIGURE 7:**
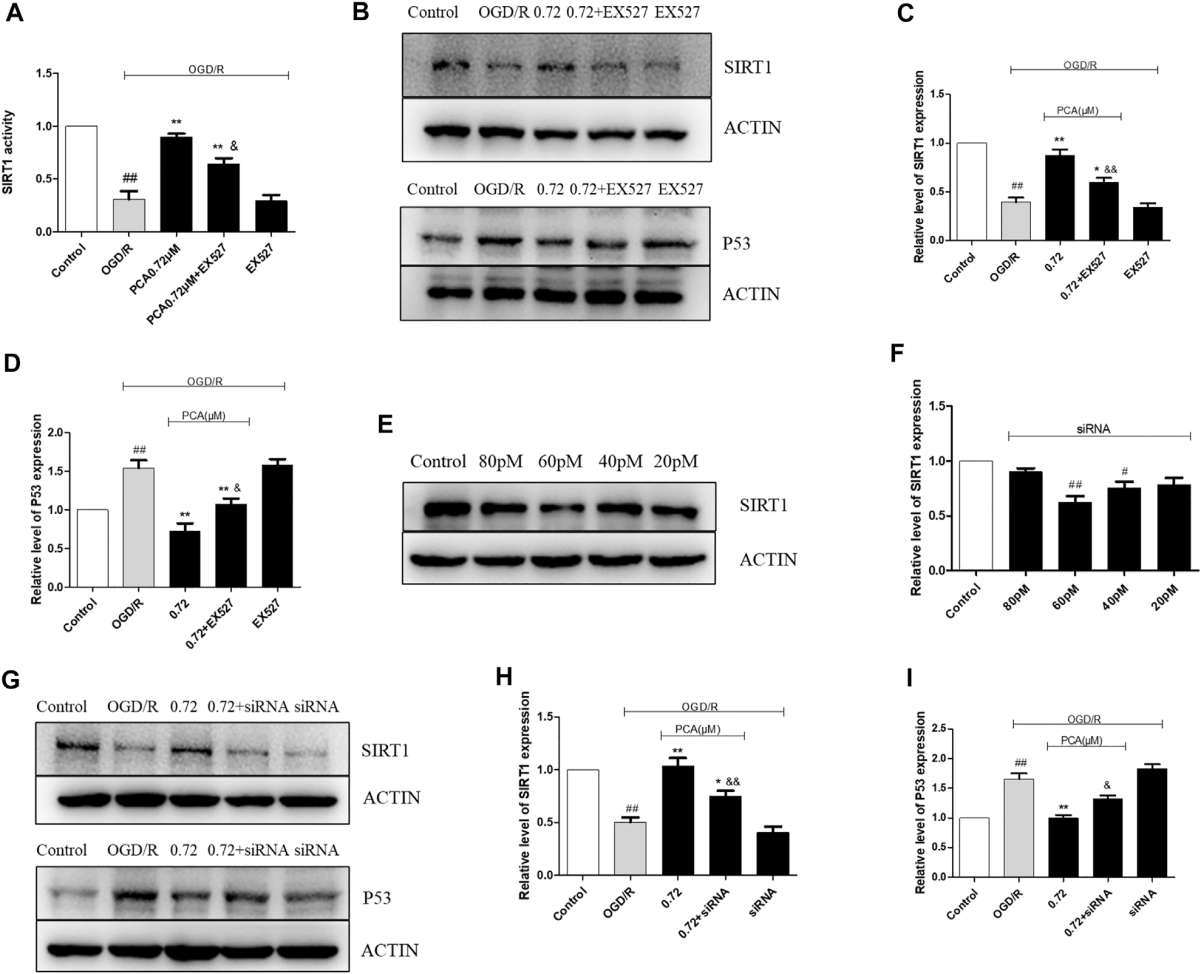
The effect of EX527 and SIRT1 siRNA on SIRT1 and P53. **(A)** The effect of EX527 on SIRT1 activity after OGD/R-induced injury. **(B–D)** Representative images of WB analysis and the semi-quantification of SIRT1, P53 in HUVECs. **(E, F)** Expression levels of SIRT1 proteins under different concentrations of SIRT1 siRNA (20–80 p.m.). **(G–I)** Representative images of WB analysis and the semi-quantification of SIRT1, P53 in HUVECs. The values are expressed as the mean ± SD (*n* = 3). ^##^ *p* < 0.01, ^#^ *p* < 0.05 vs. Control, ^**^ *p* < 0.01, ^*^ *p* < 0.05 vs. OGD/R, ^&&^ *p* < 0.01, ^&^ *p* < 0.05 vs. PCA 0.72 μM.

The appropriate concentration of EX527, an inhibitor of SIRT1 deacetylase activity, was determined to be 10 μM (*p* < 0.05) (Figures 6E,F). Then, the results showed that EX527 reduced the expression level and activity of SIRT1, which was abolished by PCA (0.72 μM) treatment (*p* < 0.05) (Figures 7A–C). Furthermore, the results indicated that EX527 treatment abolished the enhanced effect of PCA on P53 expression in OGD/R-induced HUVECs (*p* < 0.05) (Figure 7D). Similar results were obtained when SIRT1 expression was silenced by 60 pM SIRT1 siRNA (*p* < 0.01) (Figures 7E–I).

### SIRT1 Mediated the Anti-Oxidative Effect of PCA in OGD-Injury HUVECs

**FIGURE 8:**
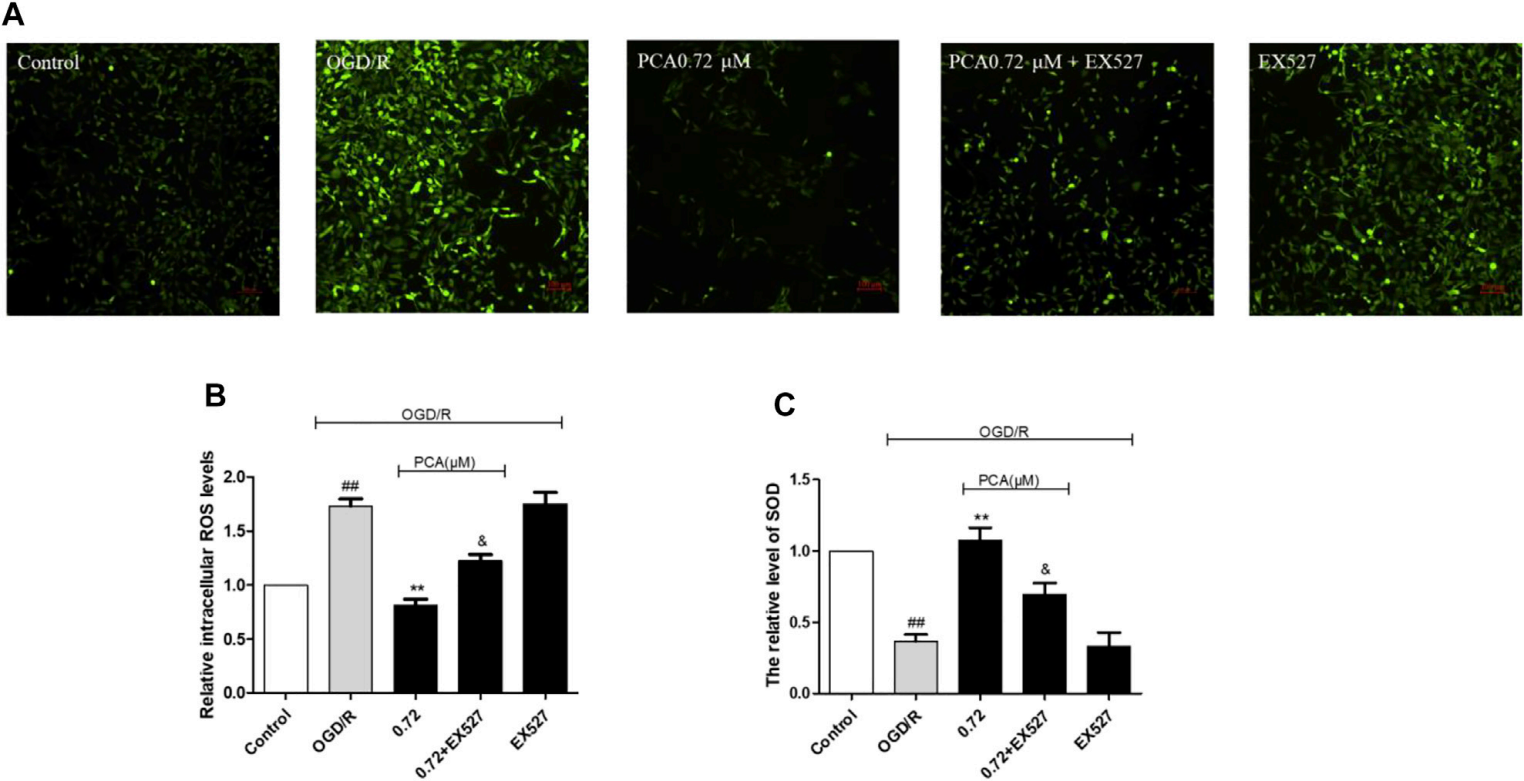
Inhibition of SIRT1 by EX527 attenuates the antioxidant activity of PCA. **(A, B)** Quantitation of HUVECs ROS level was shown. **(C**) Quantitation of HUVECs SOD activity was shown. The values are expressed as the mean ± SD (*n* = 3). ^##^ *p* < 0.01 vs. Control, ^**^ *p* < 0.01 vs. OGD/R. ^&^ *p* < 0.05 vs. PCA 0.72 μM.

Compared with the group treated with PCA only (0.72 μM), treatment with EX527 + PCA resulted in a significant increase in ROS level (Figures 8A,B) and a decrease in SOD level (Figure 8C) (*p* < 0.05). This indicated that EX527 could eliminate the antioxidant effect of PCA.

### SIRT1 Mediated the Suppression Effect of PCA on Apoptosis

**FIGURE 9:**
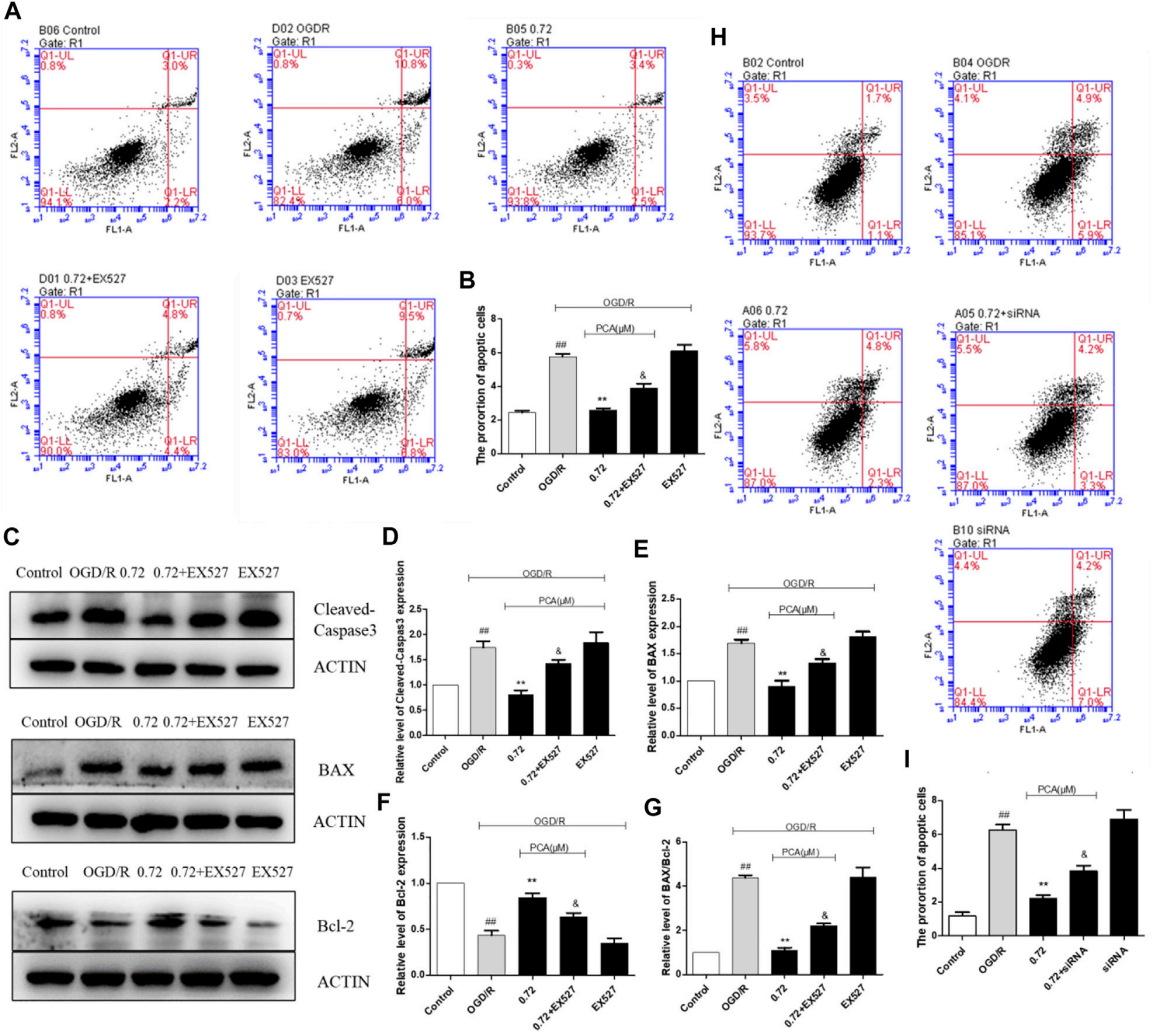
Inhibition of SIRT1 by EX527 attenuates the anti-apoptotic effect of PCA. **(A, B)** Representative images of flow cytometry analysis of EX527 influence and quantification of cell apoptosis rate. **(C–G)** Representative images of WB analysis and the semi-quantification of Cleaved-Caspase3, BAX and Bcl-2 in HUVECs. **(H, I)** Representative images of flow cytometry analysis of siRNA influence and quantification of cell apoptosis rate. The values are expressed as the mean ± SD (*n* = 3). ^# #^ *p* < 0.01 vs. Control, ^**^ *p* < 0.01 vs. OGD/R, ^&^ *p* < 0.05 vs. PCA 0.72 μM.

Flow cytometry analysis showed that when EX527 was added to block SIRT1 activity, the inhibition effect of PCA on apoptosis was attenuated, and rate of apoptosis increased from 2.5% to 4.4% (*p* < 0.05) (Figures 9A,B). Results of apoptosis-related protein levels also showed that EX527 could abolish the anti-apoptosis effect of PCA (*p* < 0.05) (Figures 9C–G). SIRT1 siRNA also attenuated the anti-apoptotic effect of PCA (*p* < 0.05) (Figures 9H,I).

### SIRT1 Mediated the Promotion Effect of PCA on Autophagy

**FIGURE 10:**
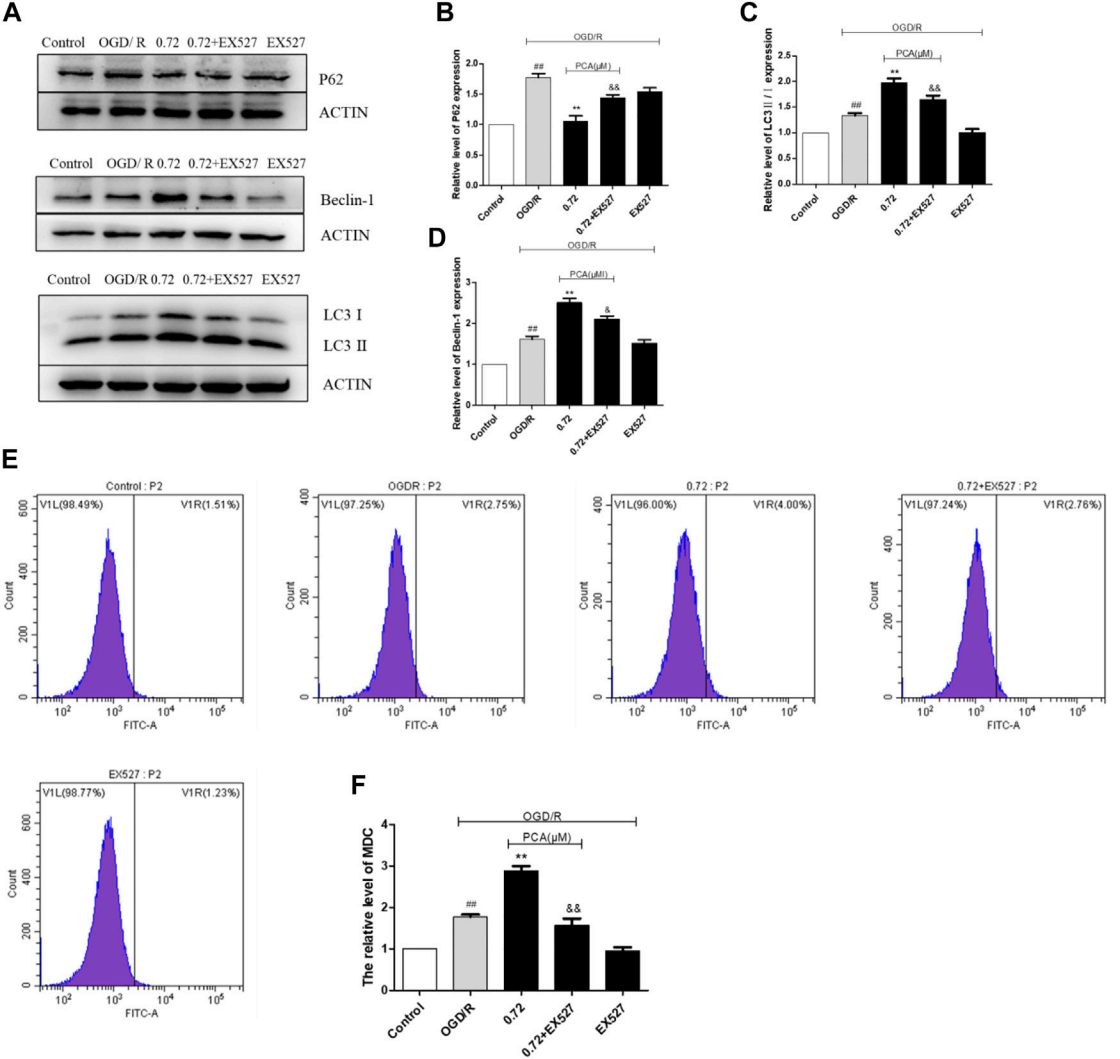
Inhibition of SIRT1 by EX527 attenuates the pro-autophagy effect of PCA. **(A–D)** Representative images of WB analysis and the semi-quantification of P62, Beclin-1 and LC3 in HUVECs. **(E, F)** Representative images of flow cytometry analysis of PCA influence and quantification of cell autophagy rate. The values are expressed as the mean ± SD (*n* = 3). ^##^ *p* < 0.01 vs. Control, ^**^ *p* < 0.01 vs. OGD/R, ^&&^ *p* < 0.01, ^&^ *p* < 0.05 vs. PCA 0.72 μM.

**FIGURE 11:**
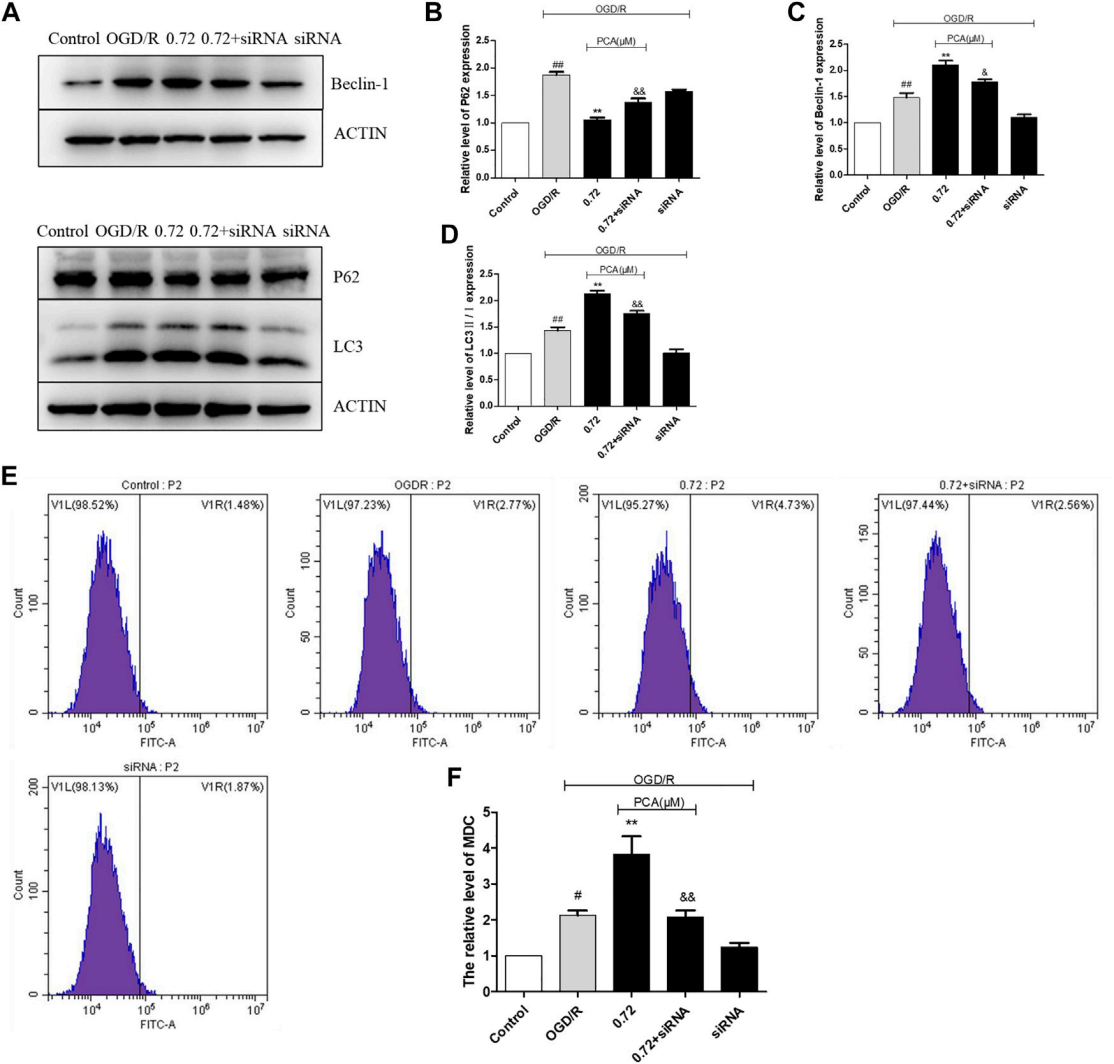
Inhibition of SIRT1 by siRNA attenuates the pro-autophagy effect of PCA. **(A–D)** Representative images of WB analysis and the semi-quantification of P62, Beclin-1 and LC3 in HUVECs. **(E, F)** Representative images of flow cytometry analysis of PCA influence and quantification of cell autophagy rate. The values are expressed as the mean ± SD (*n* = 3). ^##^ *p* < 0.01, ^#^ *p* < 0.05 vs. Control, ^**^ *p* < 0.01 vs. OGD/R, ^&&^ *p* < 0.01, ^&^ *p* < 0.05 vs. PCA 0.72 μM.

The results indicated that EX527 could attenuate the effect of PCA on the expression level of autophagy markers P62, Beclin-1, and LC3 II/I (*p* < 0.05) (Figures 10A–D). In addition, MDC staining results indicated that EX527 could also abolish the pro-autophagy ability of PCA (*p* < 0.05) (Figures 10E,F). Likewise, SIRT1 siRNA also attenuated the pro-autophagic effect of PCA, similar to the effect of EX527 (Figures 11A–F).

## Discussion

As an important and well-known traditional Chinese medicine, *Salvia miltiorrhiza Bunge* has been widely used to treat various vascular disease including cardiovascular diseases worldwide. PCA has been confirmed to be the major bioactive component from *Salvia miltiorrhiza Bunge* against cardiovascular ischemic injury. However, the potential mechanisms of PCA on cardiovascular ischemic injury is largely unexplored. Our study focused on the effect and mechanism of PCA in the treatment of ECs after OGD/R injury, which can provide more exhaustive theoretical support for *Salvia miltiorrhiza Bunge* in the traditional treatment of cardiovascular disease and ischemic stroke.

The occurrence of ischemic disease is related to the coexistence of multiple complex factors, such as inflammation, oxidative stress, blood**-**brain barrier disorder, platelet activation, and cell apoptosis (Ge et al., 2019; Navis et al., 2019; Sarkar et al., 2019; Ren et al., 2022). In recent years, most of the studies on PCA in the treatment of ischemic diseases have been limited to a single factor, such as pyrolysis (Guo et al., 2022), mitochondrial energy metabolism (Zeng et al., 2021), apoptosis (Wan et al., 2021), or oxidative stress (Guo et al., 2017). To investigate the impact of PCA on ischemic diseases more extensively, our study proposes a novel mechanism by which PCA alleviates OGD/R-induced injury to HUVECs by promoting autophagy and inhibiting apoptosis through SIRT1 axis.

Autophagy is a catabolic process of intracellular degradation, that is involved in cell survival, development, and death (Zhang T et al., 2022). Autophagy, as an adaptive response, exerts a key role in cellular homeostasis and maintenance (Ren et al., 2019). Apoptosis is a process of programmed cell death, which can be triggered by hypoxia, ROS, and stress (Zhou et al., 2022). Recent studies have shown that autophagy is closely related to apoptosis. Zhou et al. (2022) showed that the root extract of *Scutellaria baicalensis Georgi* promoted β-cell function and prevented apoptosis in diabetes treatment by inducing autophagy. Luo et al. (2019) showed that SIRT1 promoted autophagy *via* AMPK activation and reduced hypoxia-induced apoptosis, to protect cardiomyocytes from hypoxic stress. It is widely accepted that autophagy exhibits a protective role by inhibiting apoptosis and our research showed the similar results.

BAX is considered to be a pro-apoptotic protein that activates Caspase3 to induce cell apoptosis (Dong et al., 2021; Wu et al., 2022). Bcl-2 acts as an anti-apoptotic gene, overexpression of Bcl-2 has been reported to prevent cerebral ischemic damage (Zhao et al., 2003), and the ratio of BAX/Bcl-2 is of great significance in determining apoptosis (Wu et al., 2022). It has also been shown that excessive accumulation of ROS and reduction of SOD can also cause apoptosis (Cai et al., 2021). Previous studies have found that OGD/R-induced injury can trigger apoptosis (Cao et al., 2018), which is consistent with our results. Regulation cell apoptosis is paramount for cell survival during OGD/R-induced injury (Tian et al., 2016). Our study also found that PCA may exert pharmacological effects by inhibiting apoptosis. In addition, the results of cell flow cytometry also proved this conclusion.

LC3 is a widely used autophagosome marker and the ratio of LC3 II/I can be used to indicate the level of autophagy in cells (Zhang L et al., 2022). Beclin-1 initiates the nucleation step of autophagy to initiate autophagic flux (Kang et al., 2011). P62 is a multifunctional protein that is, linked to autophagosome-localized protein LC3 during autophagy, and mediates the degradation of autophagosomes (Lin et al., 2012). This means that when autophagy occurs, LC3 expression increases while P62 expression decreases. Therefore, autophagy dysfunction is usually manifested by the accumulation of P62 (Katsuragi et al., 2015). In our study, the expression of P62 was increased, suggesting that autophagy was disturbed after OGD/R-induced injury and the same results were found by Ren et al. (2019). The mechanism of autophagy is complex and often has different effects on different diseases. Activation of autophagy by oxidative stress, cellular starvation, and hypoxia, tends to improve the survival rate of vascular ECs (Li et al., 2018). In addition, it has been demonstrated that the modulation of autophagy could confer protection against IR injury (Zhang et al., 2017). Our research also supports this conclusion, showing that PCA act to promote autophagy.

P53 acts as a main orchestrator of cellular responses to different types of stress, regulates cell cycle arrest, apoptosis, DNA repair, and genetic stability (Laptenko and Prives, 2006). Previous studies have demonstrated that P53 is involved in neuronal death that occurs following ischemia (Endo et al., 2006). Increased expression of P53 is often accompanied by apoptosis (Cai et al., 2021), and autophagy can block the induction of apoptosis by inhibiting the activation of P53 and caspases (Thorburn, 2008; White, 2016). Together, these lines of evidence suggest that P53 is involved in the regulation of autophagy and apoptosis, as confirmed by the increased expression of P53 following OGD/R-induced injury in this study. SIRT1 is an NAD^+^-dependent deacetylase involved in the regulation of inflammation, oxidative stress, autophagy, DNA damage repair, cell apoptosis, and cellular senescence (Zeng et al., 2019; Guo et al., 2021). In recent studies, it was found that SIRT1 could promote autophagy and inhibit apoptosis, thereby attenuating radiation-induced IEC-6 cell damage and preventing hypoxic heart damage (Luo et al., 2019; Qin et al., 2021). Furthermore, Ren et al. (2019) reported that appropriate up-regulation of SIRT1 expression could promote autophagy and inhibit apoptosis in OGD/R-induced injury, thereby improving cellular tolerance to OGD/R injury. In our study, PCA can promote the expression of SIRT1 and reduce the expression of P53, but when SIRT1 is inhibited, the expression of P53 increases, and the anti-apoptotic and pro-autophagic abilities are lower than that of PCA treatment. Therefore, our studies confirm that PCA can rescue OGD/R-induced HUVECs injury by promoting autophagy and inhibiting apoptosis through the SIRT1 axis.

## Conclusion

The current study shows that PCA can enhance antioxidant activity, inhibit apoptosis, and promote autophagy to rescue OGD/R-induced HUVECs injury through the SIRT1 axis. Oxidative stress and the resulting cell death are major risk factors for IR injury-related diseases, and PCA is significantly affected in these conditions. Therefore, given the potential broad utility of PCA therapy, future studies are warranted to further clarify the role of PCA in ischemic diseases and its mechanisms involved.

## Data Availability

The original contributions presented in the study are included in the article/supplementary material, further inquiries can be directed to the corresponding authors.
